# Notes

**Published:** 1899-08-26

**Authors:** 


### The Institutional Workshop.

### NOTES.

The Sanitary Institute has arranged a course of lectures and demonstrations for sanitary officers during September, October, and November. Many names well known in medicine, architecture, and engineering appear in the list of lecturers.

It seems a great pity that Sunday concerts should provoke such active opposition. The newly-instituted concerts at the Crystal Palace, which are being given in aid of the Prince of Wales's Hospital Fund, have been the latest to excite opposition, and legal proceedings will probably be taken by some of the inhabitants in the locality of the Palace.

The open-air treatment for consumption is becoming so popular that activity in the establishment of sanatoria is very marked. It is becoming recognised that this form of institution can be maintained on a business basis so as to produce a profit. A company is now being formed to open a sanatorium at Bournemouth, called the Stourfield Park Sanatorium, to be utilised by the richer classes.

A new departure is proposed at Birmingham in connection with the Hospital Saturday Fund. This is the formation of a consultative institution, which practically is intended to consist of a list of consultants who will see contributors to the Hospital Saturday Fund amongst the working classes for the reduced fee of ten shillings. The medical men of Birmingham have objected to the formation of a list, although they have expressed willingness to see deserving cases at a reduced fee. The Birmingham workman, however, does not care to be considered a deserving case, and declines to have his position inquired into.

The autumn congress of the Sanitary Institute commences on August 29th and closes on September 2nd. On Wednesday conferences will be held in the morning. On Thursday the papers and discussions will be devoted to sanitary science and preventive medicine, engineering and architecture, physics, chemistry, and biology; and in the evening Mr. Malcolm Morris will lecture on tuberculosis. On Friday the meetings of sections will be continued; a closing general meeting will be held; and in the evening Bailie J. Dick, J.P., will lecture on "The Glasgow Infectious Diseases Hospitals."

Three poor-law unions in and around Liverpool have appointed a joint committee to deal with the tuberculosis question as it affects the paupers under their care, and this committee has agreed to request the Toxteth guardians to undertake the erection of the proposed hospital, the other boards undertaking to pay their share of the cost. Such an arrangement reminds us of the inception of the Metropolitan Asylums Board, which in origin was arranged for the better carrying out of matters common to the various boards of guardians in London, and we feel tempted to ask, Will the treatment of the consumptives of London be the next little duty thrown on this ever-willing Board?

A cottage hospital has been erected at Watton, Norfolk, in commemoration of the Jubilee. Mrs. Arnald de Grey presented the site, and Mrs. Perkins has contributed £1,100 towards an endowment fund.

Among the amenities of life in the United States is to be reckoned the delight of being involved in a tramway strike. During one of these pleasing functions at Cleveland, Ohio, according to the New York *Medical Record*, the strikers organised a boycott with such effect that the chemists refused to make up the prescriptions of physicians who rode in the cars while visiting their patients.

Next year is to be a year of congresses in Paris, it being expected that more than 100 international congresses of various kinds will be held there during the time that the Exposition will remain open. A large number of those which are likely to be of interest to medical men will centre around the date of the International Medical Congress. This will take place between August 2nd and August 9th, and will be immediately followed by the International Congress of Hygiene. At the same time will be held the International Congress of Experimental and Therapeutic Hypnotism, so that it will be possible to mingle the serious problems of sanitation with studies in lighter vein.

